# Photochromic properties of three 2D MOFs based on 1-carboxyethyl-4,4′-bipyridinine†

**DOI:** 10.1039/c9ra06703e

**Published:** 2019-10-17

**Authors:** Jinjian Liu, Jing Li, Wenbo Lu

**Affiliations:** Key Laboratory of Magnetic Molecules & Magnetic Information Materials Ministry of Education, The School of Chemical and Material Science, Shanxi Normal University Linfen 041004 China liujj@sxnu.edu.cn

## Abstract

Viologen units have been widely used to impart metal–organic frameworks (MOFs) with photochromic properties. However, construction of a stable photochromic system in viologen MOFs has not been fully explored. Herein, we report three examples of MOFs, namely, {[Cd(CEbpy)(*m*-BDC)(DMF)]·2H_2_O}_*n*_ (1), {[Cd(CEbpy)(*p*-BDC)(H_2_O)]·H_2_O}_*n*_ (2), and {[Zn(CEbpy)(*p*-HBDC)(*p*-BDC)_0.5_]·H_2_O}_*n*_ (3) based on benzenedicarboxylic acids (*m*-H_2_BDC = 1,3-benzenedicarboxylic acid, *p*-H_2_BDC = 1,4-benzenedicarboxylic acid) and a viologen-derived ligand 1-carboxyethyl-4,4′-bipyridine (L = CEbpy). As expected, the incorporation of the viologen unit into the frameworks results in the predefined photochromism upon both sunlight and UV-light. Compounds 1–3 feature a two-dimensional (2D) layered structure and are all photochromic due to the formation of CEbpy radicals by photoinduced electron transfer (PET). The aggregates build an interesting stable crystalline framework that exhibits long-lived color constancy in the solid state.

## Abstract 2

Viologen units have been widely used to impart metal–organic frameworks (MOFs) with photochromic properties.

## Graphical abstract

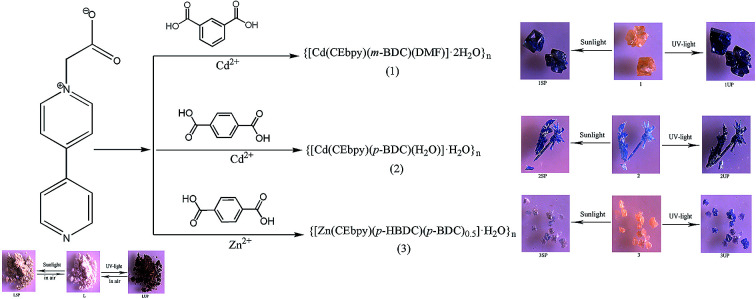

## 1. Introduction

As a unique highly ordered and porous material, metal–organic frameworks (MOFs)^1^ have become increasingly popular due to their configurable structure, high surface area and adjustable porosity. Because of the adjustability and multifaceted modularity of MOFs, the functional organic moiety can be self-assembled, grafted or embedded to create a multifunctional MOF that has been used for gas storage/separation, catalysis, proton conduction and sensing.^2^ Photochromic materials allow for convenient visual monitoring due to photoinduced color changes, which may be accompanied by changes in other properties.^3^ Recently, as a kind of functional material, photochromic MOFs have attracted more and more attention because they can display attribute changes immediately by light irradiation without any cumbersome processing.^4^ It is feasible to synthesize such functional MOFs using a photoactive organic linker.

It is well known that viologen/4,4′-bipyridinium derivatives have electron acceptability and redox activity.^5^ Because of the excellent electron acceptability and Lewis acidic sites, viologens are extensively studied in the field of chromic materials such as photochromism, thermochromism and electrochromism.^6^ The viologen cation (V^2+^) is capable of undergoing electron transfer (ET) to form a viologen radical (V˙^+^) and can generally be accompanied by an identifiable color change.^7^ The photoinduced electron transfer (PET) process occurs without bond cleavage/formation, thereby exerting little disturbance to the molecules and crystal structures, which facilitates reversible photochromism in the solid state.^8^ In addition, it is known that viologen derivatives are excellent photochromic organic ligands providing controllability of substituents on the N atom of the pyridinium ring, which can promote the production of photosensitive MOFs those have chemical stability and redox activity.^9^ Viologen derivatives are usually selected as the organic ligands to be introduced into MOFs to construct new photochromic materials due to their excellent characteristics,^10^ which have been reported as photochromic materials several times.^11^

Viologens are well known to be easily photo-reduced accompanied by obvious color changes.^12^ Furthermore, the photochromic behaviors can usually be observed under anaerobic conditions or a flash-photolytic scale, because the reduced radicals are highly sensitive to oxidation.^13^ However, the instability of organic radicals in the air remains a challenge for applications. To date, stable organic radicals obtained have been limited to a few systems with variable color behavior.^14^ The preparation of cross-linked viologen MOFs is reported to be a good way to perform PET and to generate stable free radicals in air, accompanied by color changes.^15^ By limiting air oxidation and reverse ET, photogenerated free radicals are stabilized by crystal packing, which undergoes diffusion control in the crystalline state. So far, to construct a stable photochromic system in viologen MOFs has not been fully explored.^16^

In this example, we propose a viologen-derived ligand, 1-carboxyethyl-4,4′-bipyridinine (L = CEbpy) with the terminal oxygen and nitrogen atoms serving as coordination sites, in which long-lived color species by embedding the viologen units into the condensed MOFs can be prepared. Three metal–viologen MOFs, formulated as {[Cd(CEbpy)(*m*-BDC)(DMF)]·2H_2_O}_*n*_ (1), {[Cd(CEbpy)(*p*-BDC)(H_2_O)]·H_2_O}_*n*_ (2), {[Zn(CEbpy)(*p*-HBDC)(*p*-BDC)_0.5_]·H_2_O}_*n*_ (3) (*m*-H_2_BDC = 1,3-benzenedicarboxylic acid, *p*-H_2_BDC = 1,4-benzenedicarboxylic acid) are prepared. Furthermore, the photochromism of the three MOFs are investigated. As anticipated, under the irradiation of UV-light and sunlight, compounds 1–3 exhibit noticeable color changes due to the production of CEbpy radicals. It is notable that the photoinduced free radicals of the three MOFs can keep stable in air for months at room temperature.

## 2. Experimental section

### 2.1 Materials and instruments

All reagents and solvents were commercially purchased and used without further purification. 1-Carboxyethyl-4,4′-bipyridine (L = CEbpy) was synthesized according to the literature;^17^ Elemental analyses (C, H, and N) were conducted on a Vario EL III CHNOS elemental analyzer; X-ray powder diffraction (PXRD) data were obtained with a Rigaku Ultima IV-185 diffractometer; UV-vis diffuse reflectance spectra were performed at room temperature with a Varian Cary 5000 UV-visible spectrophotometer; electron paramagnetic resonance (EPR) spectroscopy were recorded at room temperature on a Bruker A300-10/12 electron resonance spectrometer; a ThermoFisher ESCALAB 250 X-ray photoelectron spectrometer (powered at 150 W) by Al Kα radiation (*λ* = 8.357 Å; spot size, 500 m) was used to perform X-ray photoelectron spectroscopy (XPS).

#### Preparation of 1

A mixture of Cd(NO_3_)_2_·4H_2_O (30.8 mg, 0.1 mmol), CEbpy (21.4 mg, 0.1 mmol) and *m*-H_2_BDC (16.7 mg, 0.1 mmol) was heated to dissolve in a mixed solvent of 10 mL water, 5 mL methanol and 5 mL *N*,*N*-dimethylformamide (DMF), and filtrated. The mixture was kept undisturbed in air at room temperature for 10 days, and then yellow crystals were obtained with a yield of 26% (based on CEbpy). Elemental analysis: anal. calcd for C_23_H_25_CdN_3_O_9_: C, 46.05; H, 4.21; N, 7.01%. Found: C, 45.91; H, 4.27; N, 7.14%. IR (KBr, cm^−1^): 3443, 1651, 1567, 1392, 1211, 1121, 1019, 878, 840, 744, 614, 512.

#### Preparation of 2

This procedure was similar to the synthesis of 1, except that *p*-H_2_BDC (16.7 mg, 0.1 mmol) was used instead of *m*-H_2_BDC (16.7 mg, 0.1 mmol). Light blue crystals were obtained with a yield of 28% (based on CEbpy) for 6 days. Elemental analysis: anal. calcd for C_20_H_18_CdN_2_O_8_: C, 45.60; H, 3.45; N, 5.32%. Found: C, 45.81; H, 3.51; N, 5.27%. IR (KBr, cm^−1^): 3436, 1642, 1560, 1385, 1218, 1123, 1011, 872, 841, 749, 616, 516.

#### Preparation of 3

This procedure was similar to the synthesis of 2, except that Zn(CH_3_COO)_2_·2H_2_O (22.0 mg, 0.1 mmol) was replaced by Cd(NO_3_)_2_·4H_2_O (30.8 mg, 0.1 mmol). Yellow crystals were obtained with a yield of 32% (based on CEbpy) for 7 days. Elemental analysis: anal. calcd for C_24_H_19_ZnN_2_O_9_: C, 52.91; H, 3.52; N, 5.14%. Found: C, 52.93; H, 3.59; N, 5.26%. IR (KBr, cm^−1^): 3426, 1627, 1554, 1371, 1234, 1178, 1102, 870, 854, 767, 715, 616, 524.

**Scheme 1:**
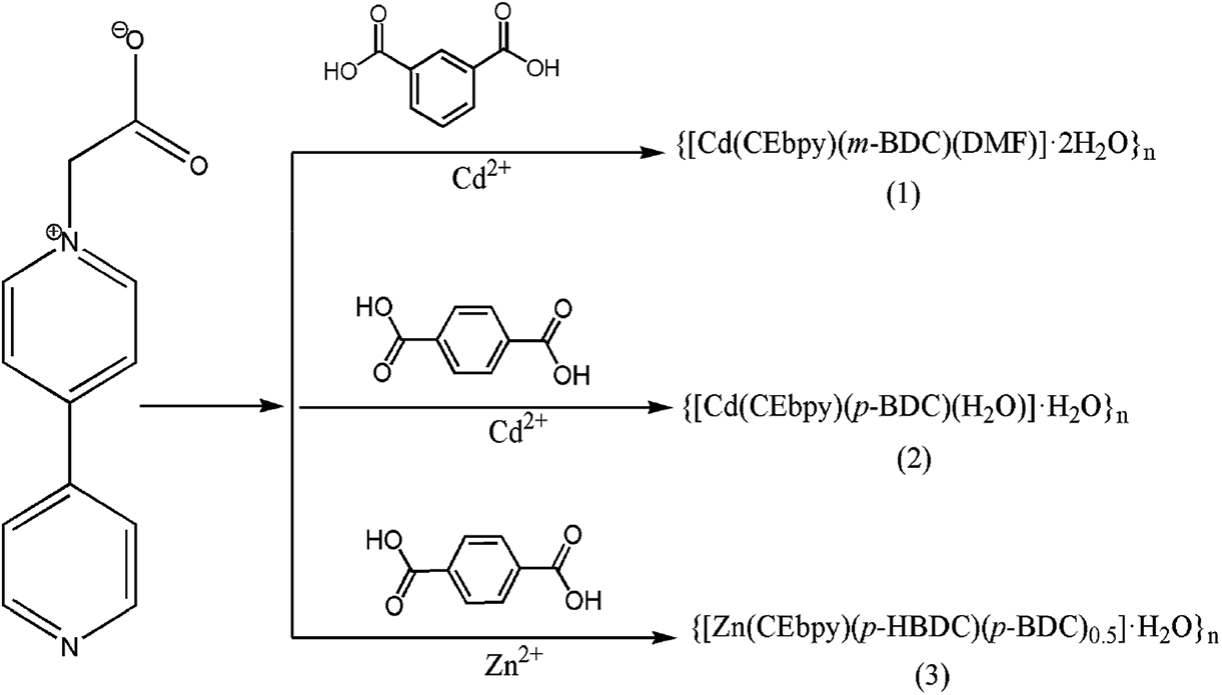
Preparation of compounds 1–3.

The powder X-ray diffraction experimental spectra of the synthesized three MOFs synthesized are in good agreement with the simulated samples, confirming the solid phase purity (Fig. S1†) (Scheme 1).

### 2.2 X-ray crystallography

Crystallographic data were recorded at 293 K using graphite monochrome Mo-Kα (*λ* = 0.71073) on an Oxford Gemini diffractometer for X-ray diffraction data of compounds 1–3. Empirical absorption correction of spherical harmonics was implemented in the SCALE3 ABSPACK scaling algorithm.^18^ The SHELXTL-97 crystallographic software package was used to solve and refine the structures solved by direct method and refined on *F*^2^ by full matrix least squares techniques.^19^ All non-hydrogen atoms were refined anisotropically. The crystallographic data of 1–3 is listed in Table 1, and the selected bond lengths and bond angles are listed in Table S1.†

**Table 1:** Crystal data and structure refinement for 1–3 at 293 K^a^^b^

Compound	1	1UP	2	3
CCDC code	1956829	1956570	1902518	1918048
Empirical formula	C_23_H_25_CdN_3_O_9_	C_23_H_25_CdN_3_O_9_	C_20_H_18_CdN_2_O_8_	C_24_H_19_ZnN_2_O_9_
Formula weight	599.87	599.87	526.76	544.80
Crystal size (mm)	0.4 × 0.4 × 0.3	0.3 × 0.3 × 0.3	0.34 × 0.33 × 0.3	04 × 0.3 × 0.3
Crystal system,	Monoclinic	Monoclinic	Triclinic	Monoclinic
Space group	*P*2_1_/*c*	*P*2_1_/*c*	*P*1̄	*P*2_1_/*c*
*a* (Å)	10.624(9)	10.5787(6)	9.6740(6)	7.4557(5)
*b* (Å)	16.457(13)	16.3960(16)	10.0787(6)	19.1019(13)
*c* (Å)	14.415(11)	14.3239(13)	11.7148(7)	15.6785(10)
*α* (°)	90	90	96.8800(10)	90
*β* (°)	110.510(19)	110.304(2)	92.3220(10)	93.186(2)
*γ* (°)	90	90	114.9100(10)	90
Volume (Å^3^)	2361(3)	2330.1(4)	1023.23(11)	2229.4(3)
*Z*	4	4	2	4
*D*_c_ (g cm^−3^)	1.679	1.701	1.710	1.566
*F*(000)	1204	1204	1056	1072
m (mm^−1)^	0.984	1.997	1.118	1.156
*R*_1_/w*R*_2_, [*I* ≥ 2*σ*(*I*)]^a^^,^^b^	0.0552, 0.1662	0.0515, 0.1589	0.0336, 0.0927	0.0525, 0.1735
*R*_1_/w*R*_2_, (all data)	0.0597, 0.1755	0.0546, 0.1644	0.0374, 0.1089	0.0578, 0.1797

a R_1_ = ∑‖*F*_o_| − *F*_c_‖/∑*F*_o_|

b wR = [∑w(*F*_o_^2^ − *F*c^2^)]/∑w(*F*_o_^2^)^2^]^1/2^

## 3. Results and discussion

### 3.1 Structural description

**Fig. 1:**
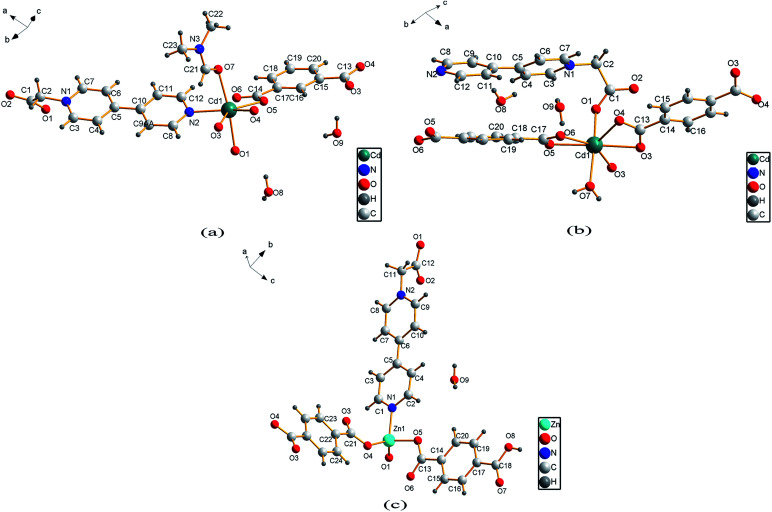
Ball-and-stick graph representation of the asymmetric unit of 1 (a), 2 (b) and 3 (c).

**Fig. 2:**
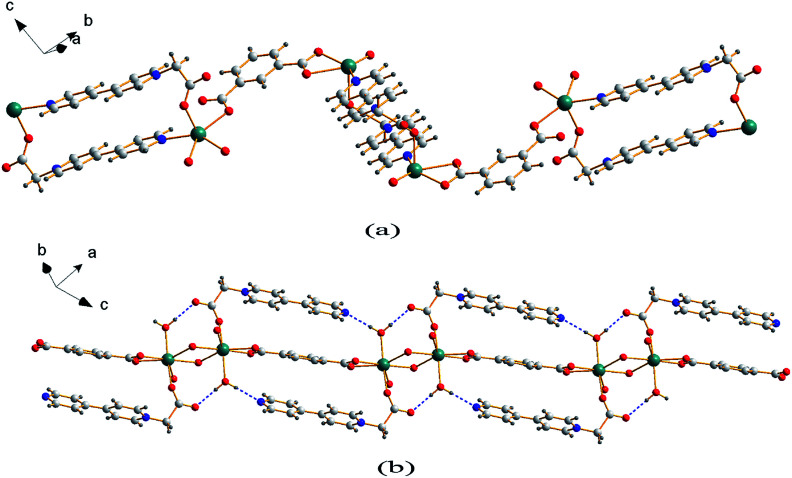
1D coordination chain of 1 (a) and 2 (b) based on the CEbpy ligand, benzenedicarboxylic acids and Cd^2+^ ion.

**Fig. 3:**
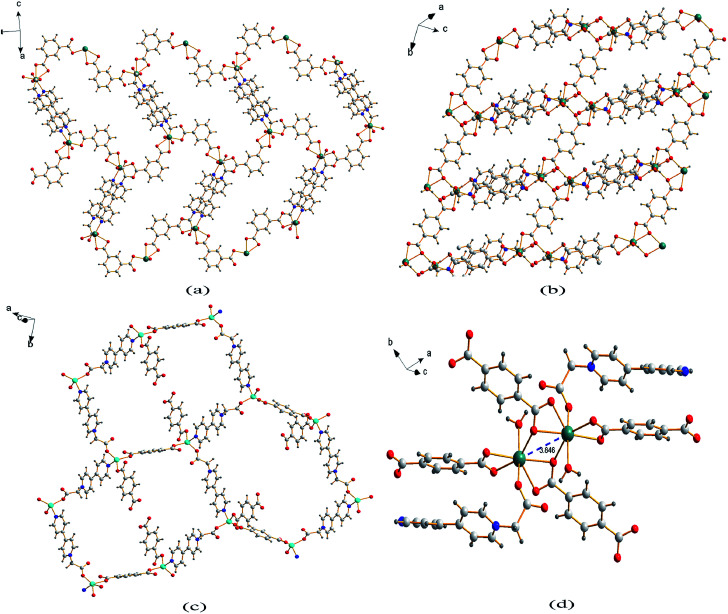
2D coordination layer of 1 (a), 2 (b) and 3 (c); (d) the Cd_2_ molecular building block with a Cd⋯Cd separation for 2.

Single crystal X-ray analysis reveals that compound 1 is a two-dimensional (2D) MOF crystallizing in the monoclinic space group *P*2_1_/*c*. As shown in Fig. 1a, the asymmetric unit of 1 contains one Cd^2+^ cation, one CEbpy ligand, one *m*-BDC^2−^ ligand, one DMF ligand and two lattice water molecules. Every Cd^2+^ cation is six-coordinated in a distorted octahedral geometry by five O atoms (*d*_Cd–O_ = 2.221 − 2.475 Å) from one CEbpy ligand, two *m*-BDC^2−^ ligands, one DMF ligand and one N atom (*d*_Cd–N_ = 2.304 Å) from another CEbpy ligand. There are two different coordination modes in the two carboxylate groups of the *m*-BDC^2−^ ligand, one is monodentate, while the other is bidentate. The carboxylate group of the CEbpy ligand is monodentate. The coordinated DMF molecule acts as a terminal ligand to complete the octahedral coordination environment of the Cd^2+^ ion. As illustrated in Fig. 3a, the coordination interaction among the two bis-CEbpy groups (Fig. 2a), four *m*-BDC^2−^ ligands and six coordinated Cd^2+^ ions makes compound 1 a 2D network with (6, 3) topology. Strong π–π stacking interactions between the pyridine rings of CEbpy ligands in the layer exist with the center–center distance of 3.737 Å (Fig. S2a†), and C–H⋯O, O–H⋯O hydrogen bonds are observed (Table S2†), which will well stabilize the crystal structure.

Compound 2 crystallizes in the triclinic space group *P*1̄ and also features a 2D framework structure. The asymmetric unit of 2 consists of one Cd^2+^ ion, one CEbpy ligand, two half *p*-BDC^2−^ ligands, one coordinated water and two half lattice water molecules (Fig. 1b). Each Cd ion shows a slightly distorted pentagonal bipyramid structure, coordinated by five O atoms from three different *p*-BDC^2−^ ligands, one O atom from the CEbpy ligand and one O atom from the coordinated water. The Cd–O distances vary from 2.273 Å to 2.414 Å. Unlike the 2-connected node of CEbpy in compound 1, it acts as a 1-connected node in 2, the N atom does not participate in coordination, but forms a stronger hydrogen bond O(7)–H⋯N(2) (2.040 Å) with the coordinated water molecule, which also forms another strong hydrogen bond O(7)–H⋯O(2) (1.887 Å) with the carboxylate O atom not involved in the coordination in the CEbpy ligand on the other side (Fig. 2b). One prominent structural feature of 2 is the presence of a binuclear Cd_2_ unit Cd_2_(CO_2_)_6_(H_2_O)_2_. The *p*-BDC^2−^ linker with four carboxylate O atoms in the μ-4 mode links with four Cd^2+^ ions to form two Cd_2_ clusters with a Cd⋯Cd separation of 3.846 Å (Fig. 3d). Each Cd_2_ cluster can be viewed as a second building unit (SBU) surrounded by four *p*-BDC^2−^ ligands with bidentate chelating modes. The binuclear unit is further connected to adjacent ones by a *p*-BDC^2−^ ligand and two CEbpy ligands to form a 1D three rows of horizontal chain network (Fig. 2b). The adjacent chains are further linked by *p*-BDC^2−^ ligands to form a 2D layer (Fig. 3b). The 2D layers are stacked by face-to-face π⋯π stacking interactions (the center–center distance is 3.764 Å, Fig. S2b†) and hydrogen bonds to form the supramolecular array.

Compound 3 crystallizes in the monoclinic space group *P*2_1_/*c*, and also possesses a 2D framework structure with (6, 3) topology like compound 1. The asymmetric unit of 3 consists of one Zn^2+^ cation, one CEbpy ligand, one coordinated *p*-HBDC^−^ ligand, a half coordinated *p*-BDC^2−^ ligand and one lattice water molecule (Fig. 1c). Each Zn ion is bound by one O atom from the *p*-HBDC^−^ ligand, one O atom from the *p*-BDC^2−^ ligand, one O atom from the CEbpy ligand and one N atom from another CEbpy ligand, showing a distorted tetrahedron coordination mode. Each Zn ion connects CEbpy ligands and *p*-BDC^2−^ ligands to form a 2D grid-like layered motif (Fig. 3c). The bond lengths of Zn–O vary from 1.953 Å to 1.980 Å, and the bond length of Zn–N is 2.078 Å in the structure. The two carboxyl groups in the *p*-BDC^2−^ ligand both participate in the coordination. However, only one carboxyl group in the *p*-HBDC^−^ ligand participates in the coordination. Every CEbpy ligand adopts a bidentate coordination mode bridging two Zn^2+^ cations, which is the same as 1, but different from 2.

### 3.2 Photochromism

Because of the presence of the electron-deficient viologen moiety, the photochromic behaviors of the three MOFs are investigated.^20^ Compounds 1–3 are found sensitive to both sunlight and UV-light and undergo photochromic transformations upon irradiation at room temperature in air.

**Fig. 4:**
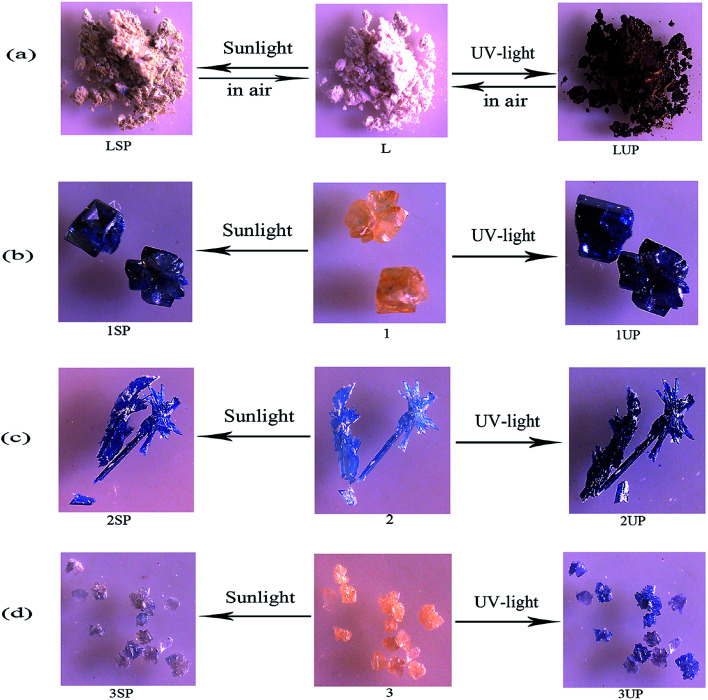
Photographs before and after irradiation for the CEbpy ligand (a), 1 (b), 2 (c) and 3 (d).

Compound 1 is photosensitive, and exhibits a visible color change from yellow to dark blue (1SP) when exposed to sunlight for 5 min in air at room temperature, and after 20 min of irradiation, the coloration is completely saturated. Compound 1 also exhibits a visually detectable change from yellow to darker blue (1UP) within 2 min (Fig. 4b) upon UV-light irradiation (Hg lamp, 365 nm, 175 W). The similar photochromic behaviors can also be found in 2 and 3. As shown in Fig. 4c, for 2, a color change from light blue to blue (2SP) upon sunlight irradiation after 10 min and to dark blue (2UP) upon UV-light irradiation after 1 min. Compound 3 undergoes a slower color change from yellow to brown (3SP) upon sunlight irradiation after 40 min, and turns to blue (3UP) upon UV-light irradiation after 5 min (Fig. 4d). It should be noted that, there is also a color change for the ligand CEbpy during irradiation, indicating that the ligand CEbpy alone exhibits photochromic behaviors. As shown in Fig. 4a, white crystals of CEbpy turn to khaki (LSP) after being irradiated with sunlight for 1 h, and turn to dark brown (LUP) upon UV-light irradiation after 15 min. After exposure to sunlight, the photoproduct of CEbpy is fully decolored in the dark after one day, and the photoproduct upon UV-light irradiation takes two days to decolorize. However, all the three MOFs based on the CEbpy ligand are more stable in air. Unlike the ligand CEbpy, the photoproducts of the three MOFs can keep stable in the dark for a long period of time before they return to the original states. (compound 1, 25 days after sunlight irradiation to decolorize, 76 days after UV-light irradiation; compound 2, 94 days after sunlight irradiation, over six months after UV-light irradiation; compound 3, 15 days after sunlight irradiation, 62 days after UV-light irradiation). It shows that the preparation of cross-linked viologen MOFs is a good method to undergo PET and generate stable radicals in the solid state in air at the room temperature. The decolorization processes for the photoproducts of the three MOFs can be also accomplished by heating the samples at 140 °C for 4–6 hours. These colorization–decolorization processes can be repeated at least for six cycles by alternating light irradiation and heating treatment, showing good reversibility.

**Fig. 5:**
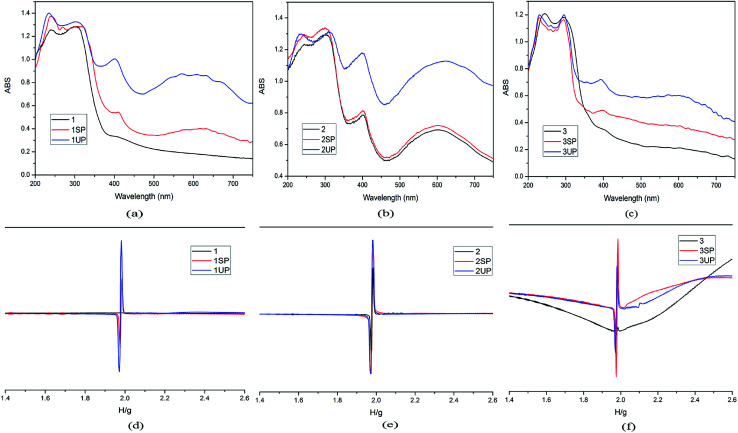
UV-vis spectra (a for 1, b for 2, c for 3) and EPR spectra (d for 1, e for2, f for 3) before and after irradiation for the three MOFs.

In order to clarify the photochromic processes, the solid UV-vis reflectance and EPR spectra of the three MOFs before and after irradiation are investigated. As shown in Fig. 5a and c, the UV-vis spectra of photoproducts (1SP, 1UP, 3SP, 3UP) show two new bands at about 410 nm and 610 nm compared to origin samples 1 and 3 after sunlight and UV-light irradiation. However, it is found that the UV-vis reflectance spectrum of the original sample 2 has the absorption bands similar to the photoproducts of 1 and 3. The absorption bands get enhanced after irradiation with sunlight and UV-light, (Fig. 5b). The difference in the UV-vis reflectance spectra for the three MOFs should be due to the fact that 2 is more sensitivity upon indoor-light than compounds 1 and 3. The viologen moiety is known to be redox-active and can generate radicals under light irradiation. Based on the previous works on the viologen derivatives,^21^ we can conclude that the color change of the three MOFs should be due to the production of CEbpy radicals. EPR spectra confirm this radical generation. For compounds 1 and 3, there is no EPR signal observed before irradiation, and strong radical signals of *g* = 2.0037 occur after irradiation (Fig. 5d and f). For compound 2, a strong EPR signal at *g* = 2.0037 has already been observed due to the sensitivity to indoor-light, and the EPR signals became stronger after sunlight and UV-light irradiation (Fig. 5e). At the same time, the single crystal XRD data and PXRD patterns indicate that the photochromism is unrelated to photolysis or photoinduced isomerisation (Table 1 and Fig. S1†).^22^ The XPS test of 1 before and after UV-light irradiation is used to identify the PET process. As shown in Fig. S3a and b,† the core energy level spectra of Cd 3d and C 1s are almost the same before and after irradiation. However, the variation of those of O 1s and N 1s are discernible. The O 1s core level spectrum has a transition to higher binding energy from 530.58 eV to 530.88 eV (Fig. S3c†), suggesting that the O atoms lose electrons. Different from the case of O 1s, the N 1s peaks shift to lower energy (Fig. S3d†). This result indicates that photochromism of 1 should originate from ET from the carboxylate O to the N atom of the pyridinium ring.

It is well known that there are many factors affecting the photochromic behavior of the viologens, the main factor is the electron transfer distance between the electron-rich group and the electron-deficient bipyridinium unit.^23^ In compounds 1–3, the electron donors are the carboxyl O atoms of CEbpy ligands and benzenedicarboxylic acids to provide electrons to the pyridinium N atoms. Structural analysis of the shortest ET distances for the three MOFs reveals that the O1⋯N1 distance is 2.767 Å and the O1⋯N1⋯C2 angle is 60.39° in compound 1, the O1⋯N1 distance is 2.709 Å and the O1⋯N1⋯C2 angle is 60.78° in compound 2, the O2⋯N2 distance is 2.717 Å and the O1⋯N1⋯C2 angle is 60.31° in compound 3. These values favor the interaction between the carboxylate group donor and the viologen acceptor unit.^24^ The tight condensed packing mode provides an opportunity for intramolecular ET and creates a viologen radical with color changes. The ET distances of the three compounds are much shorter than those commonly reported (>3.0 Å),^25^ thus it may be the reason that why the three compounds are not only sensitive to UV-light but also sensitive to sunlight. Compound 2 is also found sensitive to indoor-light, rather than compounds 1 and 3. By analyzing and comparing the interactions around the pyridine rings of the three compounds, this phenomenon is mainly caused by the following three reasons: firstly, the distance (2.709 Å) between the carboxylate O and the pyridinium N in 2 is shorter than 1 (2.767 Å), and similar to 3 (2.717 Å); secondly, comparing the structures of 2 and 3, compound 2 forms a strong π–π stacking interaction between the pyridine rings of CEbpy ligands, promoting the ET progress, while in 3, there is no π–π stacking interaction; at last, compared to 3, the interactions of the O(7)–H⋯N(2) hydrogen bonds between the CEbpy ligand and the coordinated water molecules in 2 may also improve the ET.

## 4. Conclusions

In summary, three MOFs {[Cd(CEbpy)(*m*-BDC)(DMF)]·2H_2_O}_*n*_ (1), {[Cd(CEbpy)(*p*-BDC)·(H_2_O)]·H_2_O}_*n*_ (2), {[Zn(CEbpy)(*p*-HBDC)(*p*-BDC)_0.5_]·H_2_O}_*n*_ (3) based on a viologen-derived ligand 1-carboxyethyl-4,4′-bipyridine (CEbpy) and benzenedicarboxylic acids (*m*-H_2_BDC = 1,3-benzenedicarboxylic acid, *p*-H_2_BDC = 1,4-benzenedicarboxylic acid) have been synthesized and characterized. The introduction of the carboxylate-viologen ligand into the closely packed MOFs facilitates long-lived color constancy achieved by the condensed packing mode. Given the existence of many known stable MOFs, structural designs can provide an opportunity to create novel stable crystalline materials with various photochromic components as building blocks.

## Conflicts of interest

There are no conflicts to declare.

## Supplementary Material

RA-009-C9RA06703E-s001

RA-009-C9RA06703E-s002
