# Supplementary material for: Photochromic properties of three 2D MOFs based on 1-carboxyethyl-4,4′-bipyridinine
Source: RSC Adv. 2019 Oct 17;9(57):33155–62. doi: 10.1039/c9ra06703e (PMC9073201; doi:10.1039/c9ra06703e)
Supplement: RA-009-C9RA06703E-s001 [file RA-009-C9RA06703E-s001.pdf]

Electronic Supplementary Material (ESI) for RSC Advances.
This journal is © The Royal Society of Chemistry 2019

Photochromic properties of three 2D MOFs based on 1-carboxyethyl-4,4′-bipyridinine

Jinjian Liu*, Jing Li, Wenbo Lu

Key Laboratory of Magnetic Molecules & Magnetic Information Materials Ministry of Education, The School of Chemical and Material Science, Shanxi Normal University, Linfen 041004, China.

Table S1. Selected bond lengths (Å) and bond angles (°) for **1**–**3**.

Compound **1**			
Cd(1)–O(5)	2.221(4)	Cd(1)–N(2)	2.304(4)
Cd(1)–O(1)	2.352(3)	Cd(1)–O(4)	2.363(4)
Cd(1)–O(7)	2.376(5)	Cd(1)–O(3)	2.475(4)
O(5)–Cd(1)–N(2)	136.42(13)	O(5)–Cd(1)–O(1)	88.18(13)
N(2)–Cd(1)–O(1)	91.10(13)	O(5)–Cd(1)–O(4)	81.32(13)
N(2)–Cd(1)–O(4)	141.88(13)	O(1)–Cd(1)–O(4)	96.18(16)
O(5)–Cd(1)–O(7)	96.34(16)	N(2)–Cd(1)–O(7)	82.64(18)
O(1)–Cd(1)–O(7)	173.74(16)	O(4)–Cd(1)–O(7)	88.8(2)
O(5)–Cd(1)–O(3)	133.55(13)	N(2)–Cd(1)–O(3)	89.62(13)
O(1)–Cd(1)–O(3)	84.53(12)	O(4)–Cd(1)–O(3)	54.18(12)
O(7)–Cd(1)–O(3)	95.39(18)		
Compound **2**			
Cd(1)–O(1)	2.273(2)	Cd(1)–O(3)	2.348(2)
Cd(1)–O(3)	2.414(2)	Cd(1)–O(4)	2.404(2)

Cd(1)–O(5)	2.408(3)	Cd(1)–O(6)	2.339(2)
Cd(1)–O(7)	2.310(2)		
O(1)–Cd(1)–O(3)	86.66(10)	O(1)–Cd(1)–O(3)	94.70(9)
O(1)–Cd(1)–O(4)	86.44(10)	O(1)–Cd(1)–O(5)	82.85(10)
O(1)–Cd(1)–O(6)	103.13(11)	O(1)–Cd(1)–O(7)	164.67(11)
O(3)–Cd(1)–O(3)	72.26(9)	O(3)–Cd(1)–O(4)	124.47(8)
O(3)–Cd(1)–O(5)	100.68(9)	O(4)–Cd(1)–O(3)	53.58(8)
O(4)–Cd(1)–O(5)	132.78(9)	O(5)–Cd(1)–O(3)	172.70(8)
O(6)–Cd(1)–O(3)	132.15(9)	O(6)–Cd(1)–O(3)	151.43(9)
O(6)–Cd(1)–O(4)	83.33(9)	O(6)–Cd(1)–O(5)	55.14(10)
O(7)–Cd(1)–O(3)	90.21(9)	O(7)–Cd(1)–O(3)	81.00(9)
O(7)–Cd(1)–O(4)	108.05(11)	O(7)–Cd(1)–O(5)	90.49(10)
O(7)–Cd(1)–O(6)	84.01(10)		
Compound **3**			
Zn(1)–O(1)	1.954(2)	Zn(1)–O(4)	1.953(2)
Zn(1)–O(5)	1.980(2)	Zn(1)–N(1)	2.078(2)
O(1)–Zn(1)–O(5)	112.37(11)	O(1)–Zn(1)–N(1)	100.32(11)
O(4)–Zn(1)–O(1)	122.39(12)	O(4)–Zn(1)–O(5)	106.46(11)
O(4)–Zn(1)–N(1)	117.50(11)	O(5)–Zn(1)–N(1)	94.45(10)

Table S2. The hydrogen bond lengths (Å) in **1–3**.

Compound **1**			
C(3)–H···O(1)	2.389	O(8)–H···O(2)	2.059
O(9)–H···O(2)	1.977	C(7)–H···O(7)	2.705
O(9)–H···O(4)	2.195	C(2)–H···O(4)	2.705
C(16)–H···O(5)	2.468	C(18)–H···O(6)	2.602
C(23)–H···O(6)	2.294	O(8)–H···O(7)	2.682
C(11)–H···O(8)	2.159	C(9)–H···O(9)	2.548
Compound **2**			
C(3)–H···O(1)	2.555	C(7)–H···O(2)	2.398
O(7)–H···O(2)	1.887	C(16)–H···O(3)	2.634
O(8)–H···O(4)	2.012	C(16)–H···O(5)	2.511
O(9)–H···O(6)	2.623	C(9)–H···O(7)	2.640
O(8)–H···O(9)	1.881	O(9)–H···O(9)	2.023
O(7)–H···O(2)	1.887	O(7)–H···N(2)	2.040
Compound **3**			
C(4)–H···O(2)	2.656	C(10)–H···O(2)	2.605
C(9)–H···O(6)	1.991	C(11)–H···O(6)	2.595
C(8)–H···O(7)	2.389	C(2)–H···O(9)	2.694
C(4)–H···O(9)	2.681	O(9)–H···O(2)	1.871

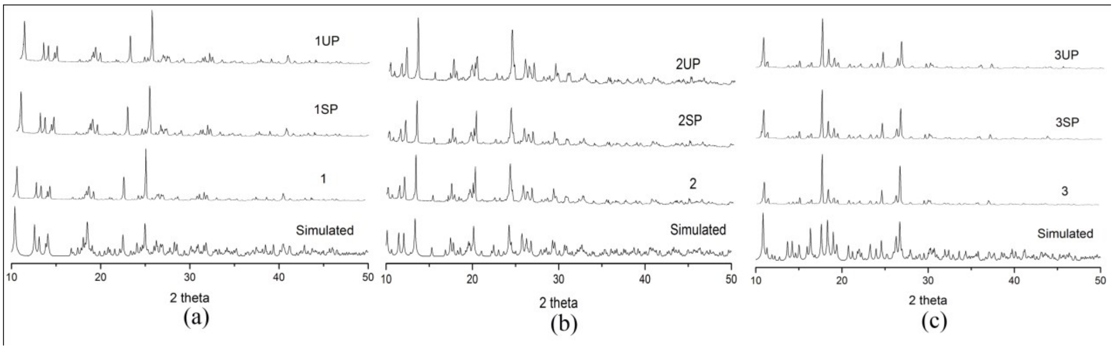

Fig. S1. The PXRD spectra before and after irradiation for **1** (a), **2** (b) and **3** (c).

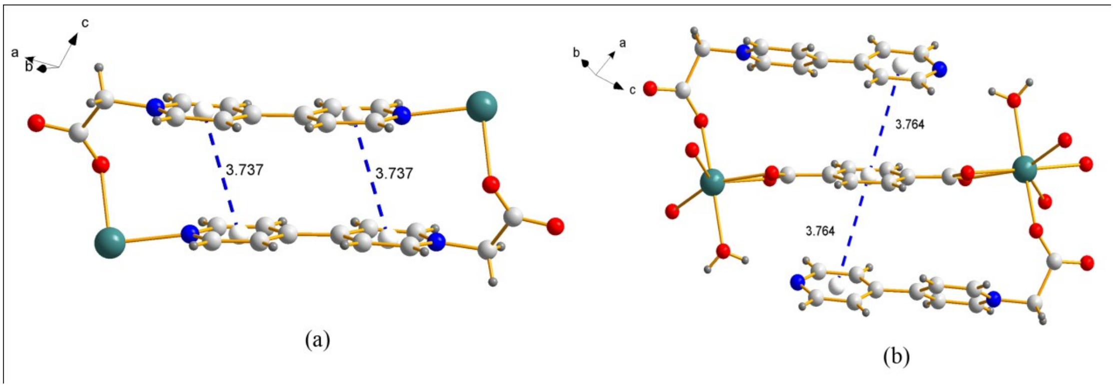

Fig. S2. π···π stacking interactions for compounds **1** (a) and **2** (b).

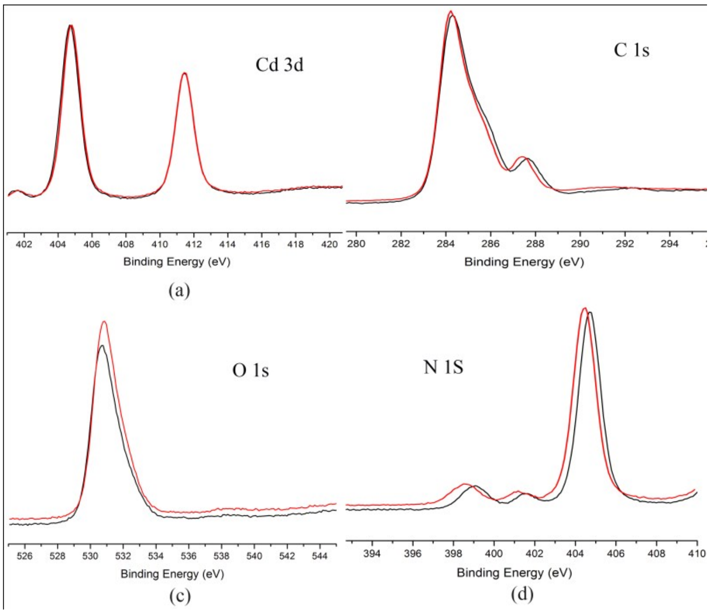

Fig.S3. XPS core-level spectra of **1** before (**1**, black line) and after UV-light irradiation (**1UP**, red line).
